# Implementation strategies in suicide prevention: a scoping review

**DOI:** 10.1186/s13012-024-01350-2

**Published:** 2024-02-26

**Authors:** Jason I. Chen, Brandon Roth, Steven K. Dobscha, Julie C. Lowery

**Affiliations:** 1 Center to Improve Veteran Involvement in Care (CIVIC), VA Portland Health Care System, U.S. Department of Veterans Affairs (VA), Portland, OR USA; 2 Department of Psychiatry, Oregon Health & Science University (OHSU), Portland, OR USA; 3 Portland VA Research Foundation, Portland, OR USA; 4 Center for Clinical Management Research (CCMR), VA Ann Arbor Healthcare System, Ann Arbor, MI USA

## Abstract

**Background:**

Implementation strategies can be a vital leveraging point for enhancing the implementation and dissemination of evidence-based suicide prevention interventions and programming. However, much remains unknown about which implementation strategies are commonly used and effective for supporting suicide prevention efforts.

**Methods:**

In light of the limited available literature, a scoping review was conducted to evaluate implementation strategies present in current suicide prevention studies. We identified studies that were published between 2013 and 2022 that focused on suicide prevention and incorporated at least one implementation strategy. Studies were coded by two independent coders who showed strong inter-rater reliability. Data were synthesized using descriptive statistics and a narrative synthesis of findings.

**Results:**

Overall, we found that studies most commonly utilized strategies related to iterative evaluation, training, and education. The majority of studies did not include direct measurement of suicide behavior outcomes, and there were few studies that directly tested implementation strategy effectiveness.

**Conclusion:**

Implementation science strategies remain an important component for improving suicide prevention and intervention implementation. Future research should consider the incorporation of more type 3 hybrid designs as well as increased systematic documentation of implementation strategies.

**Trial registration:**

< de-identified >

**Supplementary Information:**

The online version contains supplementary material available at 10.1186/s13012-024-01350-2.

Contributions to the literature

- Implementation science strategies are an important aspect of supporting the dissemination and implementation of suicide prevention interventions/programming.
- There have been limited comprehensive literature reviews characterizing implementation strategies in suicide prevention.
- Several implementation strategies were seen as more common (training and education, iterative evaluation), but there were notable gaps for those involving financial and provider support (e.g., cost sharing, financial incentives).
- Future research should consider clearer documentation of implementation strategies, more regular measurement of suicide behavior outcomes (e.g., within type 1 and type 2 hybrid studies), and direct testing of implementation strategies to inform the broader suicide prevention field.

## Background

Suicide remains a leading cause of death worldwide [1]. Although suicide rates have decreased in certain regions of the world, rates within the USA have remained elevated over the past 20 years and have continued to rise across demographic groups [1]. The Socioecological Model of Suicide Prevention posits that suicide risk is multi-factorial and impacted by factors ranging from the individual level (e.g., mental health symptoms, financial challenges) through to the societal level (e.g., health policy, stigma) [2]. Accordingly, suicide prevention and intervention programming has been developed to address risk across these levels. For example, one such multicomponent intervention approach with demonstrated effectiveness was developed through the Garrett Lee Smith Memorial Act program funded by the Substance Abuse and Mental Health Services Administration [3]. This program supports multi-component state and tribal suicide prevention initiatives to address not only those with known risk but also increase the capacity of systems to identify and support those at risk [3]. Unsurprisingly, multi-component prevention programs carry an inherent level of complexity requiring multiple strategies for implementation support. Indeed, research shows this program is effective in decreasing suicide deaths over multiple years with increased effectiveness with more years of active implementation support, highlighting the importance of implementation strategies for suicide prevention efforts [4].

Systematic reviews have identified several promising interventions for decreasing suicide attempts and deaths [5–7]. However, there remains limited adoption of these interventions as well as significant variability in effectiveness, which may be secondary to implementation challenges. A recent review identifies several implementation barriers that impact suicide prevention programming, including but not limited to high levels of complexity and cost as well as insufficient tailoring to patient needs [8]. It is, however, unknown which implementation strategies may be most helpful for addressing these needs to enhance the reach and effectiveness of promising suicide prevention programming.

In light of the need to better understand the types of implementation strategies that may enhance suicide prevention efforts, a recent systematic review attempted to describe implementation strategies used in complex interventions and determined use of such strategies was inconsistent [9]. However, this review focused only on complex suicide prevention interventions (i.e., those which had more than two components operating at different levels of intervention [e.g., individual, community]) and excluded studies focused on implementing only one intervention component (e.g., only suicide screening or suicide safety planning). However, single-component studies are common among quality improvement and implementation research projects. Its limited scope may have underrepresented the breadth of suicide prevention programming. The current scoping review expands upon this work by exploring current implementation strategies used across a broader range of suicide prevention interventions and programs.

## Methods

### Approach

The protocol for this scoping review was prospectively published online on PROSPERO (< de-identified >). A completed Preferred Reporting Items for Systematic Reviews and Meta-Analyses extension for Scoping Reviews (PRISMA-ScR) Checklist for this manuscript is available in Additional file 1. The research questions for this review were (1) what are the current implementation strategies being used for promoting suicide prevention programming as described in the literature (see “Eligibility criteria” section for further information)?; (2) how effective are these implementation strategies for promoting the use of suicide prevention programming?; and (3) What organizational factors may moderate the effectiveness of these implementation strategies? We were unable to evaluate research questions 2 and 3 due to a low volume of eligible studies and underreporting of necessary information (e.g., explicit descriptions of barriers and facilitators, site- and setting-specific information; issues identified in previous literature and discussed below) [9–11]. Additional protocol modifications, described below where applicable, included conducting two additional literature searches, suspending the USA-only eligibility criterion, implementing collaborative full-text screening, and electing to explore the studies’ usage of best practices instead of conducting a standardized quality assessment. We made these modifications to increase the inclusivity of our sample and to address challenges with the limited information present in both abstracts and full-text manuscripts.

### Searches

The search strategy (see Additional file 1) was developed in collaboration with a health sciences education and research librarian following an initial review of relevant articles (e.g., [12]). The strategy was designed to cover a broad range of topics related to suicide prevention implementation research (e.g., program development, quality improvement). Articles were obtained by searching PubMed, Scopus, PsycInfo, and the EBSCO Psychology and Behavioral Sciences Collection. The search was initially conducted in October 2019. Two additional searches were conducted in June 2021 and October 2022 due to a low volume of eligible articles from the first search.

### Eligibility criteria

To be included in the review, articles were required to have been published between January 1, 2013, and October 25, 2022 (date of the final search), be written in English, describe the implementation of a suicide prevention or intervention program (i.e., not a theory or concept paper), and describe the use of at least one implementation strategy as defined by the Consolidated Framework for Implementation Research (CFIR) [13]. Randomized controlled trials that focused only on establishing the initial effectiveness of an intervention (and not its implementation), clinical case studies, editorials, opinion pieces, newspaper articles, and other forms of popular media were excluded. During the first round of screening, reviewers decided to include studies conducted outside of the USA due to the low number of eligible studies.

### Study selection

After the removal of duplicates, two reviewers collaboratively screened the full texts of all articles for inclusion in the review. Full-text screening was used due to the limited ability to identify the use of implementation strategies from titles, abstracts, and keywords. As the use of at least one implementation strategy was required for inclusion, full-text screening was conducted collaboratively to prevent false negatives. Incongruence between reviewers was resolved by joint consensus.

### Data extraction and synthesis

The following study characteristics were initially extracted: author(s), publication year, population(s), intervention/program type, and intervention and implementation outcome(s) assessed. Data extraction was carried out primarily by one reviewer (BR) and checked for accuracy by the other (JC). Following the coding of two training studies [14, 15] to establish initial reliability, both reviewers coded implementation strategies from each article independently using a spreadsheet tool. A round of coding was conducted after each of the three literature searches. Discrepancies were resolved by joint consensus. Subsequently, reviewers collaboratively explored adherence to study conduct and reporting best practices based on the extant literature (e.g., clarity of implementation activities, assessment of implementation strategy fidelity) [11]. This protocol modification was utilized in lieu of planned quality assessment tools [16] to better fit the included studies and the implementation science context as well as the limited information available within included studies (e.g., many quality assessment domains could not be coded due to lack of information). The hybrid effectiveness-implementation study type was also determined via joint consensus at this stage based on standardized definitions from the literature [17, 18].

During implementation strategy coding, singular implementation activities that involved more than one implementation strategy were allowed to count toward all applicable strategies. CFIR implementation strategy definitions were often more granular than common narrative descriptions of study activities. For example, it was uncommon for any study to develop educational materials without distributing them. Utilizing this approach, we also sought to avoid underrepresenting strategies that commonly co-occur.

To facilitate data synthesis, reporting, and interpretation, implementation strategies were clustered based on prior publications from the Expert Recommendations for Implementing Change (ERIC) study [19, 20] (see Table 1). Clusters ranged in size from containing 3 to 17 total strategies. Revised cluster assignments (e.g., unassigned strategies, a new cluster focused on messaging-based strategies) were developed based on joint consensus.

**Table 1:** CFIR implementation strategy clusters and implementation strategies

Cluster	Implementation strategies
Adapt and tailor to context	Change service sites, promote adaptability, tailor strategies, use data experts, use data warehousing techniques
Use evaluative and iterative strategies	Assess for readiness and identify barriers and facilitators, audit and provide feedback, conduct cyclical small tests of change, conduct local consensus discussions, conduct local needs assessment, develop a formal implementation blueprint, develop and implement tools for quality monitoring, develop and organize quality monitoring system, identify early adopters, model and stimulate change, purposefully reexamine the implementation, stage implementation scale up
Utilize financial strategies	Access new funding, alter incentive/allowance structures, alter patient/consumer fees, develop disincentives, fund and contract for the clinical innovation, make billing easier, place innovation on fee for service lists/formularies, use capitated payments, use other payment schemes
Change infrastructure	Assess and redesign workflow, change accreditation or membership requirements, change liability laws, change physical structure and equipment, change record systems, change service sites, create new clinical teams, create or change credentialing and/or licensure standards, facilitate relay of clinical data to providers, mandate change, revise professional roles
Provide interactive assistance	Centralize technical assistance, implementation facilitation, provide local technical assistance
Develop stakeholder interrelationships	Build a coalition, capture and share local knowledge, create a learning collaborative, create online learning communities, develop academic partnerships, engage community resources, identify and prepare champions, inform local opinion leaders, involve executive boards, obtain formal commitments, organize clinician implementation team meetings, promote network weaving, recruit, designate, and train for leadership, use advisory boards and workgroups, use an implementation advisor, visit other sites, work with educational institutions
Support clinicians	Develop resource-sharing agreements, facilitate relay of clinical data to providers, remind clinicians
Engage consumers	Increase demand, intervene with patients/consumers to enhance uptake and adherence, involve patients/consumers and family members, prepare patients/consumers to be active participants, start a dissemination organization, use mass media
Train and educate stakeholders	Conduct educational meetings, conduct educational outreach visits, conduct ongoing training, create a learning collaborative, create online learning communities, develop an implementation glossary, develop educational materials, distribute educational materials, engage community resources, increase demand, make training dynamic, provide clinical supervision, provide ongoing consultation, shadow other experts, start a dissemination organization, use mass media, use train-the-trainer strategies
Messaging	Capture and share local knowledge, develop an implementation glossary, develop educational materials, distribute educational materials, facilitate relay of clinical data to providers, increase demand, inform local opinion leaders, intervene with patients/consumers to enhance uptake and adherence, involve patients/consumers and family members, prepare patients/consumers to be active participants, start a dissemination organization, use mass media

Clusters were adapted from Waltz et al. [19] and Perry et al. [20]. Strategy-wise cluster assignment data are available in Additional file 2

## Results

**Fig. 1:**
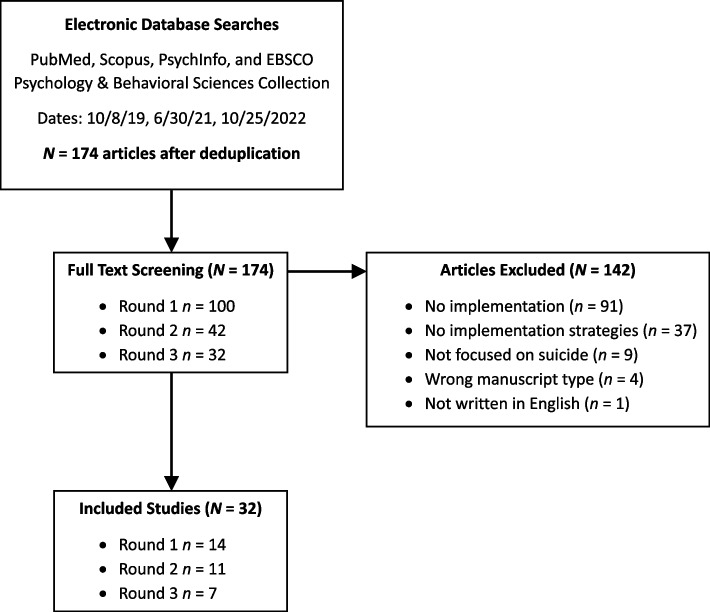
Study selection flow diagram

Following initial deduplication, full texts of 174 articles were screened. Thirty-two studies were included in the review following full-text screening [12, 14, 15, 21–49]. The most common reason for exclusion was the absence of any reported implementation activities (e.g., no intervention implemented; see Fig. 1). Study characteristics are provided in Table 2. Most studies were conducted in the USA (*n* = 26) and were single-site (i.e., implementation took place in a single organizational unit, such as one clinic; *n* = 23). Multi-site studies ranged from 3 to 65 sites. Half (*n* = 16) of the included studies described the implementation of suicide risk screening and/or risk identification, such as in settings that did not previously have such protocols. Half of the included studies utilized a hybrid effectiveness-implementation design, testing both an intervention’s effectiveness and its implementation with at least one implementation strategy [17, 18]. Of those, most (*n* = 9) were coded as type 1 hybrid effectiveness-implementation studies (i.e., focused mostly on an intervention’s effectiveness while also exploring its implementation). There were five type 2 studies (focused roughly equally on implementation and effectiveness) and two type 3 studies (focused mostly on formally testing implementation strategies while also exploring effectiveness).

**Table 2:** Coding percent agreements by batch of articles

Coding round	Strategy coding (%)
Round 1 (*n* = 14)	90.33^a^
Round 2 (*n* = 11)	87.03
Round 3 (*n* = 7)	84.89^b^
Overall (*N* = 32)	87.95^a,b^

^a^Excluding two training articles coded collaboratively [14, 15]

^b^Excluding one article coded collaboratively [25]

Intervention and implementation outcomes were not regularly distinguished by authors among the included studies. Some outcomes appeared to serve both roles depending on an intervention’s scope. For example, if training is being conducted to screen for suicide risk, training is the implementation strategy, screening is the clinical intervention, and the screening rate can be considered an implementation outcome (e.g., provider adoption) as well as a secondary intervention outcome (with patient-level suicidality the primary outcome). As such, outcomes were categorized as either intervention or implementation outcomes based on content domains to avoid misrepresenting how outcomes were used by the authors in practice.

General organizational factors outcomes (e.g., intervention adoption, costs, fidelity, leadership support) were most common (*n* = 21), followed by education- and training-related outcomes (e.g., knowledge, awareness, attitudes; *n* = 19). Studies also commonly reported effectiveness outcomes such as risk identification outcomes (e.g., screening rates; *n* = 17) and follow-up care outcomes (e.g., referral rates, appointments, psychiatric medication usage; *n* = 15). The least commonly measured were outcomes related to suicidal behavior (e.g., suicide attempts, deaths; *n* = 7) and feedback from patients (*n* = 4). Three studies provided narrative reflections on implementation processes without structured quantitative or qualitative measurement of outcomes.

Most articles adhered to at least some study conduct and reporting best practices described in the extant literature [11]. For example, most studies included some definition of their implementation outcomes (e.g., a new definition or some reference to the extant literature) and included at least some quantitative or qualitative measurement of their outcomes with clear specification of data sources (e.g., clinician feedback, electronic health record integration).

Several gaps were identified in the reporting of implementation activities. For example, several studies did not include clear implementation processes and data collection timelines (i.e., detailed enough to discern the order of events and support replication). Of the 9 multi-site studies, only Luci et al. [33] provided information on setting-level variations in the implementation process and disaggregated data by setting. Additionally, only three of the 32 included studies reported fidelity to at least one of their implementation strategies [24, 25, 34]. Overall, implementation strategies were not regularly referred to as implementation strategies (with or without citation of the ERIC framework) and were not regularly distinguished from intervention activities.

### Use of implementation strategies

Percent agreement for independent implementation strategy coding was good (see Table 2).

**Fig. 2:**
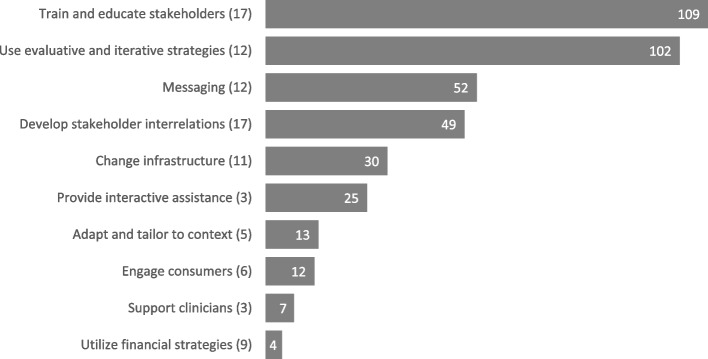
Total utilizations of strategies from each cluster across studies. Legend: Cluster sizes (number of strategies included in a cluster) are shown next to cluster names. Cluster sizes and utilization counts add to more than the total strategies and utilizations due to strategies assigned to more than one cluster (see Additional file 2)

Table 3 provides definitions, cluster assignments, and observed frequencies for all implementation strategies (i.e., the raw number of times each strategy was consensus-coded across all studies). Seventeen of the ERIC implementation strategies were not identified among the included studies. Among implementation strategies that were utilized, each was utilized 5.11 times on average (SD = 5.09) across studies suggesting studies on average employed multiple implementation strategies. ‘Purposefully reexamining the implementation’, a strategy focused on monitoring implementation progress to inform ongoing quality improvement, was most common (*n* = 20). Figure 2 shows the raw utilization of each of the individual strategies included in each cluster (i.e., sum of all individual strategy frequencies within a cluster). Strategies from the ‘train and educate stakeholders’ cluster (e.g., ‘conduct educational meetings’, ‘develop educational materials’) were utilized most often (*n* = 109). Relative to the number of strategies in each cluster (i.e., total strategy utilizations divided by cluster size), the evaluative and iterative strategies cluster (e.g., ‘purposefully reexamine the implementation’, ‘conduct local needs assessment’) cluster was the most popular.

**Table 3:** Included studies (*N* = 32), study characteristics, and total strategies coded per study (descending)

Study title	Author(s), year	Location	Intervention(s)	Population(s)	Outcome(s)	Hybrid study type^a^	Sites	Total strategies
Emergency department safety assessment and follow-up evaluation 2: An implementation trial to improve suicide prevention	Boudreaux et al. 2020 [24]	USA	Suicide risk screening, safety planning	Adults	Screening completion, suicide risk identification, suicidality outcomes, clinician screening and safety planning behaviors, attitudes, knowledge, efficacy, leadership support	2	8	23
Implementing Universal Suicide Risk Screening in a Pediatric Hospital	Sullivant et al. 2021 [45]	USA	Suicide risk screening	Youth	Screening completion, screening positivity rate, cost	N/A	1	22
Suicide on college campuses: a public health framework and case illustration	Cramer et al. 2020 [26]	USA	Campus socio-ecological suicide prevention program	Adults	Narrative reflection on the implementation process	N/A	1	19
Adapting Caring Contacts for Veterans in a Department of Veterans Affairs Emergency Department: Results From a Type 2 Hybrid Effectiveness-Implementation Pilot Study	Landes et al. 2021 [32]	USA	Caring Contacts	Veterans	Contacts completed, adoption, implementation fidelity, cost, self-directed violence, mental health service utilization, staff and Veteran qualitative feedback	2	1	19
Spreading a Strategy to Prevent Suicide After Psychiatric Hospitalization: Results of a Quality Improvement Spread Initiative	Riblet et al. 2022 [40]	USA	WHO Brief Intervention and Contact	Veterans	Program enrollment, mental health post-discharge care, Veteran satisfaction, feasibility, adherence	N/A	6	19
From Pilot to Practice: Implementation of a Suicide Risk Screening Program in Hospitalized Medical Patients	Snyder et al. 2020 [44]	USA	Suicide risk screening	Adults, Youth	Screening completion, screening positivity rate	N/A	1	16
Costs of using evidence-based implementation strategies for behavioral health integration in a large primary care system	Yeung et al. 2020 [49]	USA	Behavioral health screening (depression, self-harm, substance use)	Adults	Cost, screening completion	N/A	25	16
Harnessing Quality Improvement and Implementation Science to Support the Implementation of Suicide Prevention Practices in Juvenile Detention	Rudd et al. 2022 [41]	USA	Suicide risk screening, risk assessment, safety planning	Youth	Narrative reflection on the implementation process	N/A	1	16
Evaluation of a Suicide Prevention Training Program for Mental Health Services Staff	Donald, Dower, and Bush, 2013 [14]	Australia	Suicide prevention training	Youth	Staff knowledge, organizational links (e.g., for client referral)	2	1	15
Adapting Crisis Intervention Protocols: Rural and Tribal Voices from Montana	Belhumeur et al. 2017 [21]	USA	Crisis intervention protocol	Youth	Qualitative feedback from school-community partners	1	3	15
Personalized Implementation of Video Telehealth for Rural Veterans (PIVOT-R)	Day et al. 2021 [27]	USA	Video telehealth	Veterans	Qualitative feedback from providers, video telehealth usage	N/A	1	14
Suicide Prevention Guideline Implementation in Specialist Mental Healthcare Institutions in The Netherlands	Mokkenstorm et al. 2018 [36]	The Netherlands	Suicide prevention guidelines (documentation, assessment, safety planning, continuity of care)	Adults	Adoption, suicide rates, policy and practice changes, number of individuals trained, improved documentation, family involvement	3	24	12
Enhancing Key Competencies of Health Professionals in the Assessment and Care of Adults at Risk of Suicide Through Education and Technology	Ryan et al. 2017 [42]	Canada	Provider education (suicide awareness, risk assessment, care planning, and intervention)	Adults	Provider knowledge, confidence, and awareness	N/A	1	12
Implementation of Universal Adolescent Depression Screening: Quality Improvement Outcomes	Bose et al. 2021 [23]	USA	Depression and suicide risk screening	Youth	Screening completion, depression diagnoses, mental health referrals, psychiatric medication usage	1	1	12
Implementing Suicide Risk Screening in a Pediatric Primary Care Setting: From Research to Practice	Horowitz et al. 2022 [29]	USA	Suicide risk screening	Youth	Screening positivity rate, staff knowledge, patient and family feedback	N/A	1	12
SAVE-CLC: An Intervention to Reduce Suicide Risk in Older Veterans following Discharge from VA Nursing Facilities	Luci et al. 2020 [33]	USA	Depression screening, suicide risk assessment, care transition facilitation	Veterans	Feasibility, acceptability, frequency and promptness of contacts	1	3	10
Studying the Implementation of Zero Suicide in a Large Health Care System: Challenges, Adaptations, and Lessons Learned	Boudreaux et al. 2022 [25]	USA	Suicide risk screening, safety planning, means restriction counseling, care transition facilitation	Adults	Suicide risk identification, suicidality outcomes, uptake, clinician knowledge, clinician attitudes, clinician efficacy, screening fidelity, implementation costs, Lean evaluations	3	39	10
Development and Implementation of a Universal Suicide Risk Screening Program in a Safety-Net Hospital System	Roaten et al. 2018 [12]	USA	Suicide risk screening	Adults	Suicide risk identification	1	1	9
Suicide Concern Reporting among Utah Youths Served by a School-Based Peer-to-Peer Prevention Program	Wright-Berryman et al. 2018	USA	Peer-to-peer gatekeeper program	Youth	Suicidal ideation and behavior, program referrals, hospitalizations	N/A	65	8
Depression Screening Implementation: Quality Improvement Project in a Primary Care Clinic for First Responders	Blake, 2022 [22]	USA	Depression and suicide risk screening	Adults	Screening completion rate, average visit time, follow-up mental health appointments	N/A	1	8
Our Healthy Clarence: A Community-Driven Wellbeing Initiative	Powell et al. 2019 [39]	Australia	Multicomponent community-based intervention	Adults, Youth	Narrative reflection on implementation process	1	1	7
A Community-Based Resposne to a Suicide Cluster: A Hong Kong Experience	Lai et al. 2020 [31]	Hong Kong	Multicomponent community-based intervention	Adults, Youth	Suicide rate, qualitative feedback from working group members and participants	1	1	6
Implementation of Online Suicide-Specific Training for VA Providers	Marshall et al. 2014 [34]	USA	Online provider training	Veterans	Training completion, Veteran satisfaction, narrative reflection on implementation process	2	5	6
Quality Improvement of Pastoral Care for Major Depression in the Community of an African American Religious Organization	Garner and Kunkel, 2020 [28]	USA	Depression screening, awareness promotion	Adults	Minister knowledge and stigma, screening and referral counts	N/A	1	6
A Quality Improvement Initiative to Reduce Safety Events Among Adolescents Hospitalized After a Suicide Attempt	Noelk et al. 2019 [38]	USA	Behavioral health safety protocol	Youth	Adherence, significant safety events	1	1	5
Dialectical Behavior Therapy in College Counseling Centers: Practical Applications and Theoretical Considerations	Chugani, 2015	USA	Psychotherapy	Adults	Adoption, uptake	2	1	4
Implementation and Evaluation of the P4 Suicide Screening Tool among Sexual Assault Nurse Examiners: A Suicide Prevention and Intervention Strategy	Vaughan, 2019 [47]	USA	Suicide risk screening	Adults	Screening completion rate, mental health referrals	1	1	4
Implementation of the Signs of Suicide Prevention Program with 9th Grade Students in a Public School Setting	Tennant, 2017 [46]	USA	Peer-to-peer gatekeeper program, awareness promotion	Youth	Student knowledge, narrative reflection on implementation	N/A	1	4
Training Nursing Staff to Recognize and Respond to Suicidal Ideation in a Nursing Home	Kabatchnick, 2017	USA	Online educational module	Adults	Nursing staff knowledge and confidence	1	1	3
School Nurses Identifying At-risk Adolescents for Depression	McManus, 2020	USA	Suicide risk screening	Youth	Suicide risk identification, mental health referrals	N/A	1	3
Counseling on Access to Lethal Means-Emergency Department (CALM-ED): A Quality Improvement Program for Firearm Injury Prevention	Mueller, Naganathan, and Griffey, 2020 [37]	USA	Lethal means safety planning	Adults	Feasibility, lethal means information, and safety plans	N/A	1	3
Gatekeeper Suicide Training's Effectiveness among Malaysian Hospital Health Professionals: A Control Group Study with a Three-Month Follow-Up	Siau et al. 2018 [43]	Malaysia	Gatekeeper training program	Adults	Staff knowledge, self-efficacy, and attitudes	N/A	1	2

^a^See Landes, McBain, and Curran [18] for definitions and criteria. *N/A* = study did not utilize a hybrid effectiveness-implementation design

**Fig. 3:**
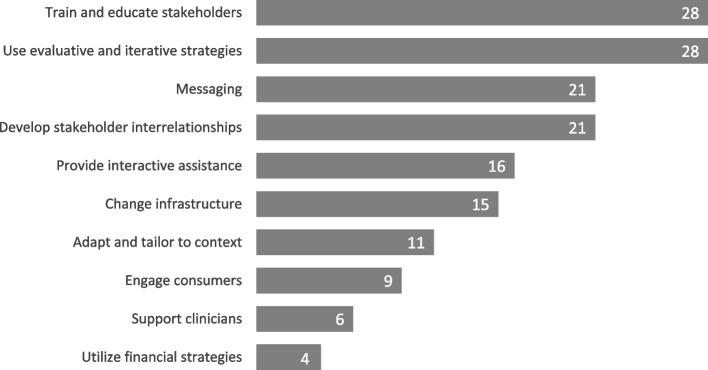
Total studies utilizing at least one strategy from each cluster

Figure 3 shows the count of studies that utilized at least one strategy from each cluster. The ‘train and educate stakeholders’ (*n* = 28) and ‘use evaluative and iterative strategies’ (*n* = 28) clusters were the most broadly used by this metric. Conversely, the ‘support clinicians’ (*n* = 6) and ‘utilize financial strategies’ *(n* = 4) clusters were used in the fewest studies. Reviewers identified the use of 10.63 implementation strategies per study on average (SD = 6.07; see Table 4 for counts per study, Additional file 2). On average, studies utilized strategies from 4.97 of the 10 strategy clusters (SD = 1.82; see Additional file 2). These results are partially attributable to frequently co-occurring strategies and strategies that belonged to more than one cluster, respectively. For example, studies that developed and evaluated a training described utilizing multiple implementation strategies that were inherent to implementing suicide prevention training (e.g., identifying barriers and facilitators, developing education materials, and making training dynamic).

**Table 4:** Implementation strategies, cluster assignments, definitions, and observed frequencies (descending)

Implementation strategy	Cluster(s)^a^	Definition^b^	Total studies
Purposefully reexamine the implementation	Use evaluative and iterative strategies	Monitor progress and adjust clinical practices and implementation strategies to continuously improve the quality of care	20
Conduct educational meetings^c^	Train and educate stakeholders	Hold meetings targeted toward educating multiple stakeholder groups (i.e., providers, administrators, other organizational stakeholders, community members, patients/consumers, families) about the clinical innovation and/or its implementation	19
Develop educational materials^c^	Train and educate stakeholders, Messaging	Develop and format manuals, toolkits, and other supporting materials to make it easier for stakeholders to learn about the innovation and for clinicians to learn how to deliver the clinical innovation. This can include technology-delivered (e.g., online/smartphone-based static or dynamic) content and health messaging	17
Distribute educational materials	Train and educate stakeholders, Messaging	Distribute educational materials (including guidelines, manuals, and toolkits) in person, by mail, and/or electronically	17
Centralize technical assistance	Provide interactive assistance	Develop and use a centralized system to deliver technical assistance focused on implementation issues	15
Conduct local needs assessment	Use evaluative and iterative strategies	Collect and analyze data related to the need for innovation	13
Use advisory boards and workgroups	Develop stakeholder interrelationships	Create and engage a formal group of multiple kinds of stakeholders to provide input and advice on implementation efforts and to elicit recommendations for improvements	12
Conduct ongoing training^c^	Train and educate stakeholders	Plan for and conduct training in the clinical innovation in an ongoing way for all individuals involved with the implementation and users of the clinical innovation, e.g., clinicians, implementation staff, practice facilitators	12
Provide ongoing consultation	Train and educate stakeholders	Provide ongoing consultation with one or more experts in the strategies used to support implementing the innovation	12
Conduct local consensus discussions	Use evaluative and iterative strategies	Include local providers and other stakeholders in discussions that address whether the chosen problem is important and whether the clinical innovation to address it is appropriate	11
Audit and provide feedback^c^	Use evaluative and iterative strategies	Develop summaries of clinical performance over a specific time period, often including a comparator, and give it to clinicians and/or administrators. Summary content (e.g., nature of the data, choice of comparator) and their delivery (e.g., mode, format) are designed to modify specifically targeted behavior(s) or actions of individual practitioners, teams, or health care organizations	11
Assess for readiness and identify barriers and facilitators^c^	Use evaluative and iterative strategies	Assess various aspects of an organization to determine its degree of readiness to implement and identify barriers that may impede implementation and strengths that can be leveraged to facilitate the implementation effort	11
Develop and organize quality monitoring system	Use evaluative and iterative strategies	Develop and organize systems and procedures that monitor clinical processes and/or outcomes for the purpose of quality assurance and improvement	10
Conduct educational outreach visits^c^	Train and educate stakeholders	Have a trained person meet with individuals or teams in their work settings to educate them about the clinical innovation with the intent of changing behavior to reliably use the clinical innovation as designed	9
Stage implementation scale up	Use evaluative and iterative strategies	Phase implementation efforts by starting with small pilots or demonstration projects and gradually move to a system-wide rollout	8
Assess and redesign workflow^c^	Change infrastructure	Observe and map current work processes and plan for desired work processes, identifying changes necessary to accommodate, encourage, or incentivize the use of the clinical innovation as designed	8
Promote adaptability	Adapt and tailor to context	Identify the ways a clinical innovation can be tailored to meet local needs and clarify which elements of the innovation must be maintained to preserve fidelity	8
Change record systems	Change infrastructure	Change records systems to allow better assessment of implementation or clinical outcomes	7
Involve executive boards	Develop stakeholder interrelationships	Involve existing governing structures (e.g., boards of directors, medical staff boards of governance) in the implementation effort, including the review of data on implementation processes	7
Mandate change	Change infrastructure	Have leadership declare the priority of the innovation and their determination to have it implemented	7
Make training dynamic	Train and educate stakeholders	Vary the information delivery methods to cater to different learning styles and work contexts, and shape the training in the innovation to be interactive	6
Conduct cyclical small tests of change	Use evaluative and iterative strategies	Implement changes in a cyclical fashion using small tests of change before taking changes system-wide. Tests of change benefit from systematic measurement and results of the tests of change are studied for insights on how to do better. This process continues serially over time, and refinement is added with each cycle	6
Involve patients/consumers and family members	Engage consumers, Messaging	Engage or include patients/consumers and families in the implementation effort	6
Identify and prepare champions	Develop stakeholder interrelationships	Identify and prepare individuals who dedicate themselves to supporting, marketing, and driving through an implementation, overcoming indifference or resistance that the intervention may provoke in an organization	6
Promote network weaving	Develop stakeholder interrelationships	Identify and build on existing high-quality working relationships and networks within and outside the organization, organizational units, teams, etc. to promote information sharing, collaborative problem-solving, and a shared vision/goal related to implementing the innovation	5
Provide clinical supervision	Train and educate stakeholders	Provide clinicians with ongoing supervision focusing on innovation. Provide training for clinical supervisors who will supervise clinicians who provide the innovation. NOTE: This should only be coded when the supervisor/trainer is an external expert or local champion with knowledge of the intervention. Having someone who is not an expert in the intervention provide supervision would not count	5
Provide local technical assistance^c^	Provide interactive assistance	Develop and use a system to deliver technical assistance within local settings that is focused on implementation issues	5
Develop and implement tools for quality monitoring	Use evaluative and iterative strategies	Develop, test, and introduce into quality-monitoring systems the right input—the appropriate language, protocols, algorithms, standards, and measures (of processes, patient/consumer outcomes, and implementation outcomes) that are often specific to the innovation being implemented	5
Develop a formal implementation blueprint^c^	Use evaluative and iterative strategies	Develop a formal implementation blueprint that includes all goals and strategies. The blueprint should include the following: (1) aim/purpose of the implementation; (2) scope of the change (e.g., what organizational units are affected); (3) timeframe and milestones; and (4) appropriate performance/progress measures. Use and update this plan to guide the implementation effort over time	5
Implementation facilitation^c^	Provide interactive assistance	A multi-faceted interactive process of problem-solving, enabling and supporting individuals, groups, and organizations in their efforts to adopt and incorporate innovations into routine practices that occurs in the context of a recognized need for improvement and a supportive interpersonal relationship	5
Access new funding	Utilize financial strategies	Access new or existing money to facilitate the implementation	4
Remind clinicians	Support clinicians	Develop reminder systems designed to help clinicians recall information and/or prompt them to use the clinical innovation	4
Tailor strategies	Adapt and tailor to context	Tailor the implementation strategies to address barriers and leverage facilitators that were identified through earlier data collection	4
Build a coalition	Develop stakeholder interrelationships	Recruit and cultivate relationships with partners in the implementation effort	3
Create a learning collaborative	Train and educate stakeholders, Develop stakeholder interrelationships	Facilitate the formation of groups of providers or provider organizations and foster a collaborative learning environment to improve implementation of the clinical innovation. NOTE: This should have a specific focus on supporting implementation and not be part of the existing protocol for a given intervention (e.g., DBT consultation groups)	3
Facilitate relay of clinical data to providers	Change infrastructure, Support clinicians, Messaging	Provide as close to real-time data as possible about key measures of process/outcomes using integrated modes/channels of communication in a way that promotes the use of the targeted innovation	3
Intervene with patients/consumers to enhance uptake and adherence	Engage consumers, Messaging	Develop strategies with patients to encourage and problem-solve around adherence	3
Use train-the-trainer strategies	Train and educate stakeholders	Train designated clinicians or organizations to train others in clinical innovation	3
Create new clinical teams	Change infrastructure	Change who serves on the clinical team, adding different disciplines and different skills to make it more likely that the clinical innovation is delivered (or is more successfully delivered)	2
Inform local opinion leaders	Develop stakeholder interrelationships, Messaging	Inform providers identified by colleagues as opinion leaders or “educationally influential” about the clinical innovation in the hopes that they will influence colleagues to adopt it	2
Create online learning communities^c^	Train and educate stakeholders, Develop stakeholder interrelationships	Create an online portal for clinical staff members to share and access resources, webinars, and FAQs related to the specific evidenced-based intervention, and provide interactive features to encourage learning across settings and teams, e.g., regular blogs, facilitated discussion boards, access to experts, and networking opportunities	2
Organize clinician implementation team meetings^c^	Develop stakeholder interrelationships	Develop and support teams of clinicians, staff, patients, and other stakeholders who are implementing or may be users of the innovation. Provide protected time for teams to reflect on the implementation progress, share lessons learned, make refinements to plans, and support one another’s learning	2
Revise professional roles	Change infrastructure	Shift and revise roles among professionals who provide care, and redesign job characteristics	2
Capture and share local knowledge	Develop stakeholder interrelationships, Messaging	Capture local knowledge from implementation sites on how implementers and clinicians made something work in their setting and then share it with other sites	1
Engage community resources^c^	Train and educate stakeholders, Develop stakeholder interrelationships	Connect practices and their patients to community resources outside the practice (e.g., state and county health departments; non-profit organizations; resources related to addressing the social determinants of health; and organizations focused on self-management techniques and support)	1
Identify early adopters	Use evaluative and iterative strategies	Identify early adopters at the local site to learn from their experiences with the practice innovation	1
Model and stimulate change	Use evaluative and iterative strategies	Model or simulate the change that will be implemented prior to implementation	1
Prepare patients/consumers to be active participants	Engage consumers, Messaging	Prepare patients/consumers to be active in their care, to ask questions, and specifically to inquire about care guidelines, the evidence behind clinical decisions, or available evidence-supported treatments	1
Recruit, designate, and train for leadership	Develop stakeholder interrelationships	Recruit, designate, and train leaders for the change effort	1
Shadow other experts	Train and educate stakeholders	Provide ways for key individuals to directly observe experienced people engage with or use the targeted practice change/innovation	1
Use data experts^c^	Adapt and tailor to context	Involve, hire, and/or consult experts to acquire, structure, manage, report, and use data generated by implementation efforts	1
Use mass media	Engage consumers, Train and educate stakeholders, Messaging	Use media to reach large numbers of people to spread the word about the clinical innovation	1
Change physical structure and equipment	Change infrastructure	Evaluate current configurations and adapt, as needed, the physical structure and/or equipment (e.g., changing the layout of a room, adding equipment) to best accommodate the targeted innovation	1
Develop academic partnerships	Develop stakeholder interrelationships	Partner with a university or academic unit for the purposes of shared training and	1
Increase demand	Engage consumers, Train and educate stakeholders, Messaging	Attempt to influence the market for the clinical innovation to increase competition intensity and to increase the maturity of the market for the clinical innovation	1
Obtain formal commitments	Develop stakeholder interrelationships	Obtain written commitments from key partners that state what they will do to implement the innovation	1
Use an implementation advisor^c^	Develop stakeholder interrelationships	Seek guidance from experts in implementation, including providing support and training for the implementation work force	1
Work with educational institutions	Develop stakeholder interrelationships	Encourage educational institutions to train clinicians in innovation	1
Alter incentive/allowance structures	Utilize financial strategies	Work to incentivize the adoption and implementation of clinical innovation	0
Alter patient/consumer fees	Utilize financial strategies	Create fee structures where patients/consumers pay less for preferred treatments (the clinical innovation) and more for less-preferred treatments	0
Change accreditation or membership requirements	Change infrastructure	Strive to alter accreditation standards so that they require or encourage use of the clinical innovation. Work to alter membership organization requirements so that those who want to affiliate with the organization are encouraged or required to use the clinical innovation	0
Change liability laws	Change infrastructure	Participate in liability reform efforts that make clinicians more willing to deliver clinical innovation	0
Change service sites	Change infrastructure, Adapt and tailor to context	Change the location of clinical service sites to increase access	0
Create or change credentialing and/or licensure standards	Change infrastructure	Create an organization that certifies clinicians in the innovation or encourages an existing organization to do so. Change governmental professional certification or licensure requirements to include delivering the innovation. Work to alter continuing education requirements to shape professional practice toward innovation bringing research skills to an implementation project	0
Develop an implementation glossary	Train and educate stakeholders, Messaging	Develop and distribute a list of terms describing the innovation, implementation, and stakeholders in the organizational change	0
Develop disincentives	Utilize financial strategies	Provide financial disincentives for failure to implement or use the clinical innovations	0
Develop resource-sharing agreements	Support clinicians	Develop partnerships with organizations that have the resources needed to implement the innovation	0
Fund and contract for the clinical innovation	Utilize financial strategies	Governments and other payers of services issue requests for proposals to deliver the innovation, use contracting processes to motivate providers to deliver the clinical innovation and develop new funding formulas that make it more likely that providers will deliver the innovation	0
Make billing easier	Utilize financial strategies	Make it easier to bill for the clinical innovation	0
Place innovation on fee-for-service lists/ formularies	Utilize financial strategies	Work to place the clinical innovation on lists of actions for which providers can be reimbursed (e.g., a drug is placed on a formulary, a procedure is now reimbursable)	0
Start a dissemination organization	Engage consumers, Train and educate stakeholders, Messaging	Identify or start a separate organization that is responsible for disseminating the clinical innovation. It could be a for-profit or non-profit organization	0
Use capitated payments	Utilize financial strategies	Pay providers or care systems a set amount per patient/consumer for delivering clinical care	0
Use data warehousing techniques	Adapt and tailor to context	Integrate clinical records across facilities and organizations to facilitate implementation across systems	0
Use other payment schemes	Utilize financial strategies	Introduce payment approaches (in a catch-all category)	0
Visit other Sites	Develop stakeholder interrelationships	Visit sites where a similar implementation effort has been considered successful	0

^a^Strategy clusters were adapted from Waltz et al. [19] and Perry et al. [20]. Remaining assignments (i.e., new messaging cluster, assigning unassigned strategies) were based on joint consensus. See Additional file 2

^b^Definitions are from Powell et al. [13] unless otherwise specified

^c^Revised definition from Perry et al. [20]

## Discussion

Overall, our review identified several current patterns in the use of implementation strategies in suicide prevention as well as several gaps in the literature. Consistent with past reviews [9, 10] few manuscripts clearly delineated or described implementation strategies in a comprehensive manner (e.g., implementation details were spread across different sections of the paper, details were limited). On average, we captured fewer strategies than those reported by Rudd and colleagues who were able to identify additional strategies through surveying authorship teams [9]. However, the most common strategy clusters noted by Rudd and colleagues were consistent with our findings. It is possible that authors were unaware they were utilizing implementation strategies and thus could not describe them in detail. As the majority of papers reviewed were not published in implementation science-oriented journals, authors may have also limited the inclusion of detailed implementation strategy information to accommodate the journal audience.

The ‘train and education stakeholders’ cluster of strategies and the ‘use evaluative and iterative strategies’ cluster were the most broadly utilized—all but four studies utilized at least one strategy from this cluster (Fig. 3). Strategies from this cluster (e.g., ‘conduct educational meetings’, ‘distribute educational materials’) were the most frequently utilized overall (Fig. 2). Similarly, education- and training-related outcomes (e.g., knowledge, awareness, attitudes), were the second most common outcome domain. This is unsurprising as the majority of suicide prevention interventions and programs focus on promoting awareness and skill-building among stakeholders [6]. However, fewer studies utilized strategies for supporting active, sustained learning (e.g., ‘provide clinical supervision’, ‘create an online learning collaborative’, ‘make training dynamic’). Additionally, 13 of the 28 studies that utilized at least one training or education strategy did not utilize any strategies from the ‘provide interactive assistance’ cluster (e.g., providing ongoing support). This is of concern, as past research shows that increased knowledge and skills from suicide training initiatives are not sustained long-term, which may, in turn, decrease the overall effectiveness of suicide prevention programming over time [4, 50].

The ‘use evaluative and iterative strategies’ cluster was also commonly reported within our sample. This is congruent with the core principles of implementation science focused on understanding and adapting to organizational contexts to enhance the adoption and maintenance of evidence-based strategies [51]. The most commonly utilized strategy from this cluster was to ‘purposefully reexamine the implementation’—a critical aspect of quality improvement emphasized across several relevant frameworks and models (e.g., Plan-Do-Study-Act [52]). Interestingly, ‘identification of early adopters’ was among the least commonly used strategies within this cluster, which may have been secondary to the limited number of studies with multiple sites in our sample.

The ‘support clinicians’ (e.g., resource sharing agreements to support clinics) and ‘utilize financial strategies’ (e.g., financial disincentives) clusters were also among the least utilized in our sample. As these strategies often require financial resources, it is possible they are more difficult to implement in light of financial challenges among healthcare systems [53]. In addition, several recent commentaries have raised concerns regarding the impact of the COVID-19 pandemic on the financial resources of hospitals, which may further limit the ability to utilize implementation strategies requiring funding [54, 55]. More popular than these strategies were those aimed toward making use of existing resources, such as those from the ‘change infrastructure’ cluster (e.g., ‘assess and redesign workflow’, ‘change record systems’) to support implementation.

Few studies reported suicide behavior outcomes. While several studies were only focused on implementation, types 1 and 2 hybrid studies remain interested in an intervention's effectiveness while exploring or formally testing its implementation and can offer vital information for informing future dissemination and implementation [17, 18]. A broader range of suicide-related outcomes would better enable such studies to evaluate whether promising interventions remain effective in practice, a key advantage of hybrid study designs. Funding agencies may wish to encourage the incorporation of Type I hybrid study procedures (e.g., qualitative inquiry on barriers and facilitators post-implementation of interventions) to ensure research studies collect sufficient information regarding implementation processes to increase future adoption and uptake of findings. Additionally, past literature has highlighted tailoring to patient needs as a key facilitator in the implementation of suicide prevention interventions [8]. However, outcomes involving feedback from patients were the least common in our sample. Similarly, the ‘engage consumers’ strategy cluster was among the least popular clusters (see Figs. 2 and 3).

Limited systematic reporting of implementation strategies and their corresponding outcomes, as well as a lack of type 3 hybrid studies (focused on formal implementation testing), limited our ability to explore the relative effectiveness of individual implementation strategies for improving suicide prevention programming (Research Question 2). Our literature search only captured studies within a 10-year period due to a desire to report on the most recent research available and excluded pertinent studies with more systematic reporting of implementation strategies outside this time period. Additionally, there was an overall low volume of multi-site studies. Among them, information necessary to explore organizational factors that could moderate the effectiveness of implementation strategies was mostly absent or unclear (e.g., specific barriers and facilitators, site-specific procedures, disaggregated data; Research Question 3).

It is possible that this information, as well as the breadth of implementation strategies, was underreported in the text of the reviewed manuscripts. Similar challenges have been reported by other reviews focused on narrower sets of suicide prevention studies [9, 10]. Rudd and colleagues found that direct outreach to authors was required to get a more comprehensive understanding of implementation science strategies present in a given study [10]. Future manuscripts may wish to utilize existing reporting guidelines, such as the Standards for Reporting Implementation Studies (StaRI) checklist in combination with frameworks that guided this review (e.g., CFIR), to help ensure implementation strategies are appropriately documented to inform the broader field [56].

## Conclusion

Implementation science remains an important and promising area of research for increasing sustainable adoption and deployment of evidence-based suicide prevention interventions and programming. Although we identified commonly used implementation science strategies and current gaps in the literature, our review was limited by the inconsistent reporting of implementation strategies within our sample. Future implementation science studies in suicide prevention should consider clearer, systematic documentation of implementation strategies utilized and associated outcomes to better inform the broader suicide prevention field. For example, journals accepting manuscripts on the implementation of suicide prevention programming may encourage the use of a common lexicon of implementation science terms or provide explicit reporting requirements. In addition, direct testing of implementation strategies through type 3 hybrid studies remains necessary to enhance the effectiveness of implementation and dissemination of suicide prevention programming.

### Supplementary Information

**Additional file 1. **Contains the Preferred Reporting Items for Systematic reviews and Meta-Analyses extension for Scoping Reviews (PRISMA-ScR) Checklist and this review’s full search strategy.

**Additional file 2. **Contains two worksheets. 1. “Coded Strategies by Study” provides the raw, consensus data from implementation strategy coding. 2. “Cluster Assignments” specifies the applicable strategy cluster(s) for each implementation strategy and whether this review changed the cluster assignment (e.g., new assignment, reassignment) from the original assignments in the literature.

## Data Availability

The datasets used and analyzed during the current study are available from the corresponding author on reasonable request.

## Abbreviations

CFIR: Consolidated Framework for Implementation Research
ERIC: Expert Recommendations for Implementing Change
